# Proteasome Inhibition Promotes Parkin-Ubc13 Interaction and Lysine 63-Linked Ubiquitination

**DOI:** 10.1371/journal.pone.0073235

**Published:** 2013-09-02

**Authors:** Grace G. Y. Lim, Katherine C. M. Chew, Xiao-Hui Ng, Adeline Henry-Basil, Roy W. X. Sim, Jeanne M. M. Tan, Chou Chai, Kah-Leong Lim

**Affiliations:** 1 Department of Physiology, National University of Singapore, Singapore, Singapore; 2 Duke-NUS Graduate Medical School, Singapore, Singapore; 3 Neurodegeneration Research Laboratory, National Neuroscience Institute, Singapore, Singapore; National Institutes of Health, United States of America

## Abstract

Disruption of the ubiquitin-proteasome system, which normally identifies and degrades unwanted intracellular proteins, is thought to underlie neurodegeneration. Supporting this, mutations of Parkin, a ubiquitin ligase, are associated with autosomal recessive parkinsonism. Remarkably, Parkin can protect neurons against a wide spectrum of stress, including those that promote proteasome dysfunction. Although the mechanism underlying the preservation of proteasome function by Parkin is hitherto unclear, we have previously proposed that Parkin-mediated K63-linked ubiquitination (which is usually uncoupled from the proteasome) may serve to mitigate proteasomal stress by diverting the substrate load away from the machinery. By means of linkage-specific antibodies, we demonstrated here that proteasome inhibition indeed promotes K63-linked ubiquitination of proteins especially in Parkin-expressing cells. Importantly, we further demonstrated that the recruitment of Ubc13 (an E2 that mediates K63-linked polyubiquitin chain formation exclusively) by Parkin is selectively enhanced under conditions of proteasomal stress, thus identifying a mechanism by which Parkin could promote K63-linked ubiquitin modification in cells undergoing proteolytic stress. This mode of ubiquitination appears to facilitate the subsequent clearance of Parkin substrates via autophagy. Consistent with the proposed protective role of K63-linked ubiquitination in times of proteolytic stress, we found that Ubc13-deficient cells are significantly more susceptible to cell death induced by proteasome inhibitors compared to their wild type counterparts. Taken together, our study suggests a role for Parkin-mediated K63 ubiquitination in maintaining cellular protein homeostasis, especially during periods when the proteasome is burdened or impaired.

## Introduction

The proteasome is a major intracellular proteolytic machinery that plays a vital role in maintaining cellular protein homeostasis through its ability to destroy unwanted proteins rapidly [1]. Proteins that are destined for proteasome-mediated degradation are usually tagged with a chain of ubiquitin linked via lysine (K) 48 through a reaction cascade that involves the ubiquitin-activating (E1), -conjugating (E2) and -ligating (E3) enzymes [2]. However, it is noteworthy that the cell is also capable of mediating alternative ubiquitin modifications such as monoubiquitination and K63-linked polyubiquitination whose roles are typically uncoupled from the proteasome [3]. For whatever reasons the proteasome becomes compromised in its function, it is difficult to imagine that the cell will continue to burden the machinery under such conditions with an endless stream of cargo proteins to be degraded. We have previously hypothesized that non-proteolytic ubiquitination of proteins may help divert proteins destined for proteasomal degradation away from the system when it becomes overwhelmed under conditions of proteolytic stress [4]. The diverted proteins, which may aggregate into inclusion bodies, are then acted upon by the complementary macroautophagy system (hereafter referred to as “autophagy”). In this way, the cell could preserve its proteasome function over prolonged periods of proteolytic stress and recover thereafter. Supporting our hypothesis, we have recently demonstrated that K63-linked polyubiquitination promotes the formation and autophagic clearance of protein inclusions [5,6].

Conceivably, the potential ability of the cell to promote K63-linked polyubiquitination during proteasomal stress would involve a dynamic partnership between relevant E3 members and Ubc13 - the only E2 known to date to mediate the formation of K63-linked ubiquitin chains [7]. Consistent with this, we have shown that over-expression of heterodimeric Ubc13/Uev1a pair alone is sufficient to promote inclusions formation and their subsequent clearance by autophagy [5]. Although the E3(s) involved remains elusive, an attractive candidate is parkin, whose mutations are associated with autosomal recessive parkinsonism [8]. We and others have demonstrated that parkin is a unique RING1-IBR-RING2-containing E3 capable of mediating multiple forms of ubiquitin modifications, including K63-linked ubiquitination [9–12]. For example, parkin can bind to UbcH7 (or H8) to mediate presumably K48-linked ubiquitin chains [13–15] or to Ubc13/Uev1a to mediate K63-linked ubiquitin chains [9,16]. Whereas Ubc13 is known to function with RING-type E3s, UbcH7 normally exhibits a preference for HECT-type E3s. The reason why parkin can work with UbcH7 is that parkin functions not as a typical RING-E3 but as a RING/HECT hybrid, i.e. it binds UbcH7 via its RING1 domain but transfer the ubiquitin through an obligate thioester-linkage via a conserved cysteine residue on its RING2 domain [17]. Notwithstanding this, the determinants that influence the choice of E2 that partner with parkin remain unclear, although we speculate that proteasomal stress may be a potential regulator. Here, we demonstrated that the recruitment of Ubc13 by parkin is dramatically and rather selectively enhanced in the presence of proteasome inhibition, which correlates with a significant increase in K63-linked polyubiquitination as detected by ubiquitin linkage-specific antibodies. Moreover, this interaction between parkin and Ubc13 can be further increased by overloading the cells with selected parkin substrates such as synphilin-1 and mutant DJ-1 (that are known to be modified by parkin-mediated K63 ubiquitination), which appears to facilitate their subsequent clearance via the autophagy route. Consistent with the proposed protective role of K63-linked ubiquitination in times of proteolytic stress, we found that Ubc13-deficient cells are significantly more susceptible to cell death induced by proteasome inhibitors compared to their wild type counterparts. Taken together, our results support a key role for parkin in maintaining protein homeostasis via K63-linked polyubiquitination during proteolytic stress.

## Methods

### Plasmids, antibodies and reagents

Plasmids expressing, HA- or myc-tagged synphilin-1, myc-tagged Siah-1 and -2, HA-tagged wild type or mutant ubiquitin, FLAG-tagged wild type or mutant parkin have been described previously [6,11,18]. The myc-tagged CHIP, UbcH6, UbcH7 and UbcH8 constructs were kind gifts from Takahashi R. (Kyoto University, Japan), while FLAG-tagged Dorfin and HA-HHARI/HA-Cbl were provided by G. Sobue (Nagoya University, Japan) and G. Guy (IMCB, Singapore) respectively. Myc-tagged Ubc13 was generated by PCR amplification using His_6_-Ubc13 as template (a gift from N. Matsuda, Tokyo Metropolitan Institute of Medical Science) and cloned into pCMV-myc vector via EcoRI and XhoI restriction sites. Untagged full length parkin was generously provided by H. Walden (London Research Institute, UK). YFP-Mitofusin2 was a gift from R. Youle (Addgene plasmid #28010). Control and Ubc13 shRNA (V2LHS_171792 and_220048) were purchased from Thermoscientific. The following mouse monoclonal antibodies were used: anti-c-myc (clone 9E10) (Roche Diagnostics, Indianapolis, IN), anti-FLAG (Sigma, St. Louis, MO, USA), anti-HA (Sigma), anti-β-actin (Sigma), anti-Ubc13 (Zymed, San Francisco, CA), anti-parkin clone PRK8 (Covance) and anti-ubiquitin clone FK1 (BIOMOL, Plymouth Meeting, PA). Linkage-specific K48 and K63 antibodies were purchased from Millipore (Temecula, CA) and BIOMOL (Plymouth Meeting, PA) respectively. Rabbit anti-GFP was purchased from Abcam (Cambridge, UK). All other reagents were purchased from Sigma, except clasto-lactacystin β-lactone (BIOMOL, Plymouth Meeting, PA) and MG-132 (A.G. Scientific, San Diego, CA).

### Cell culture, immunoprecipitation, and Western blot analysis

Human embryonic kidney (HEK) 293 cells were grown in DMEM with 10% FBS in a 5% CO_2_ atmosphere. Cells were transiently transfected with the desired plasmid(s) using the LipofectAMINE PLUS reagent (Invitrogen, San Diego, CA) according to the manufacturer’s instructions. For proteasome inhibition studies, cells were treated at 24 h post-transfection with 1 (or 2) µM MG132 for 16 h before harvesting. Sequential fractionation of transfected cell lysates into Triton-X-soluble (S) and SDS-soluble (P) fractions was carried out as previously described [18]. Immunoprecipitation from the transfected cell lysates was performed with anti-myc or anti-Ubc13 antibody and protein G PLUS/protein A-agarose (Oncogene Sciences, Uniondale, NY), and then washed five times in lysis buffer. Immunoprecipitates or cell lysates was analyzed by Western blot analysis with ECL detection reagents (Pierce Biotechnology, Rockford, IL). Wild type and Ubc13 null mouse embryonic fibroblasts (MEFs) were kind gifts from Yao T.P. (Duke University, USA) [19]. Primary MEFs from wild type and parkin null mice (exon 7 deletion) (kind gifts from Dawson T.M., Johns Hopkins Medicine, USA) [20] were generated according to published protocol [21].

### Lentivirus vector construction and cell transduction

cDNA sequences encoding myc-tagged UbcH7 and Ubc13 were PCR-amplified from their respective pcDNA3.1 plasmid clones using an EcoRV restriction site-containing forward primer (5’ AAGATATCTAGCGTTTAAA- CGGGC 3’) and a MunI restriction site-containing reverse primer (5’ AGCCAATTG- GCGGCCGCTCGAG 3’). Amplified product was digested with EcoRV and MunI and inserted into EcoRV and EcoRI sites of pL6mCWmIRESCherry. The lentivector pL6mCWmIRESCherry was modified from pLenti6/V5-D-TOPO (Invitrogen) by reengineering of the multiple cloning site, insertion of the cPPT and WPRE elements, and insertion of the IRESmCherry reporter cassette. Lentivirus packaging was performed in 293FT cells according to the protocol provided with the ViraPower™ Lentiviral Directional TOPO® Expression Kit (Invitrogen). Lentivirus particles were concentrated from cell culture supernatant according to the protocol of Deiseroth Lab (http://www.stanford.edu/group/dlab/resources/lvprotocol.pdf). Lentivirus carrying the ubiquitin expression constructs was used to transduce wild type or Ubc13 knockout MEFs. Prior to transduction, cells were cultured to ~90% confluence. Concentrated virus particles were added to cell culture medium containing 6 µg/ml of Polybrene. Long term transgene expression was maintained by selecting for resistance to Blasticidin S at a final concentration of 10 µg/ml. Transgene expression was detected by mCherry epifluorescence.

### Inclusion formation and autophagic removal

The autophagic clearance of inclusions formed under conditions of proteasomal impairment was investigated using a method originally described by Fortun et al [22]. Cells were first treated with 5 µM lactacystin to facilitate inclusion formation. After 16 h incubation, the treated cells were washed out and allowed to recover in normal media for 24 h. Concurrently, a parallel set of similarly treated cells were incubated with starvation media (1% serum) to stimulate autophagy. Thereafter, cells were processed for immunocytochemical staining for blinded evaluation of inclusions. Statistical significance for all the quantitative data obtained was analyzed using Student’s *t*-test (*P <0.05, **P < 0.001) unless otherwise stated.

## Results

### K63-polyubiquitination is enhanced in parkin-expressing cells in the presence of proteasome inhibition

Recently, K63-specific antibodies have become available from commercial sources. Although we have independently confirmed its linkage specificity in the present study (Figure S1A), we found that the sensitivity of commercially available K63 antibodies towards endogenously promoted K63 linkages under normal cell culture conditions (i.e. in the absence of proteasome inhibition) to be rather weak (not shown). To circumvent this problem, we performed our subsequent experiments in cells expressing exogenous HA-tagged wild type ubiquitin. Notably, we observed that exogenously-introduced K63 ubiquitin species (as visualized via anti-UbK63 staining) tend to reside in the pellet fraction of cell lysate (Figure S1B & C), which is consistent with our previous finding that K63-ubiquitination could influence the cellular distribution of proteins [6].

**Figure 1:**
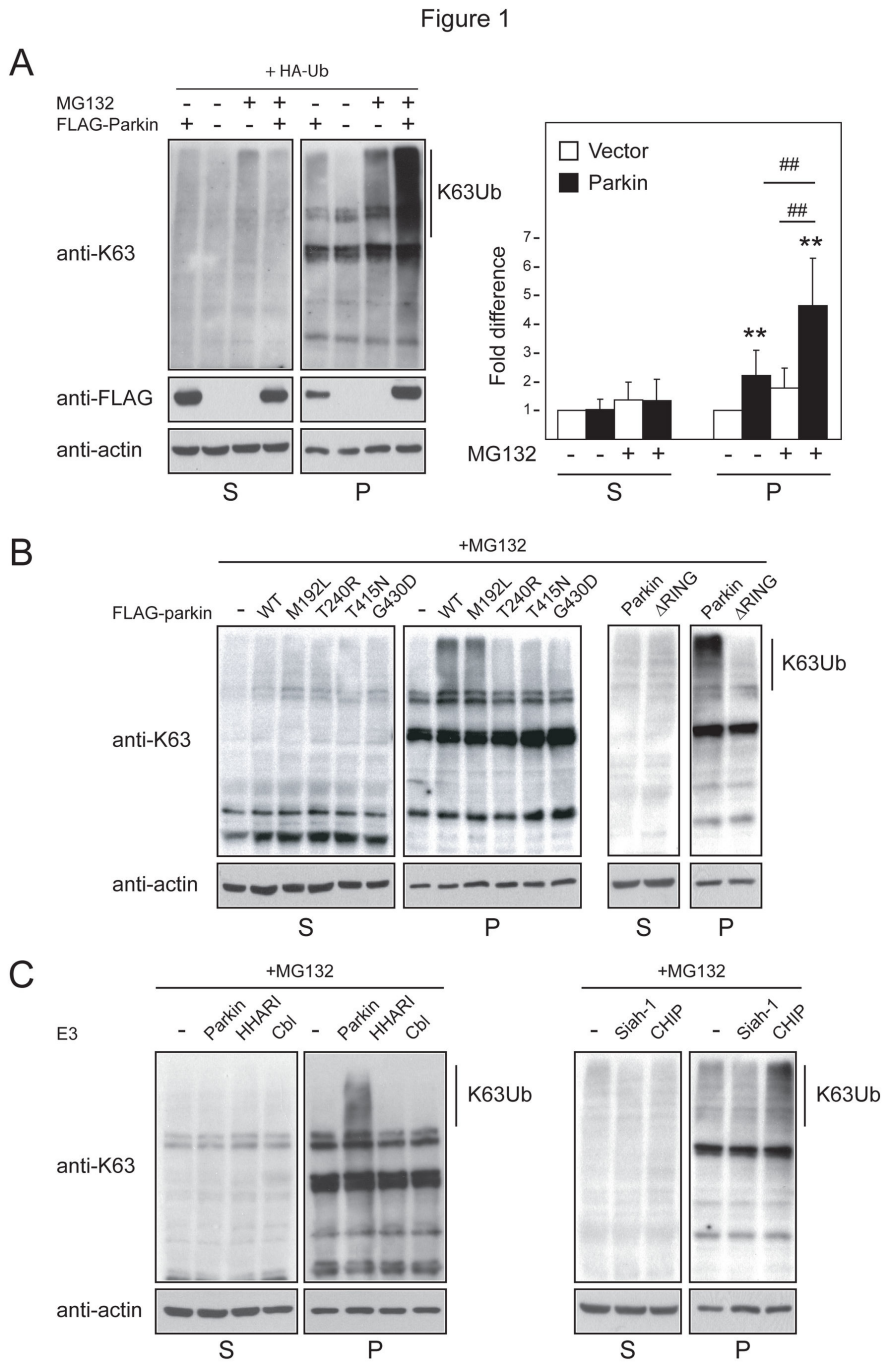
Parkin promotes K63-linked ubiquitination. (A) *Left*, Representative anti-K63 and anti-FLAG immunoblots of cell extracts sequentially prepared with Triton-X 100 (S) and SDS (P)-containing buffer from untreated or MG132-treated HEK cells transfected with HA-tagged ubiquitin, and vector or FLAG-tagged parkin, as indicated. *Right*, Bar graph showing the relative densitometric levels of K63-linked ubiqiuitination (normalized to respective actin levels) under different conditions, as indicated (**P < 0.001; ^# #^ *P* < 0.001). (B–C) Representative anti-K63 immunoblots of S and P fractions of HEK cells expressing (B) wild type or mutant parkin species, (C) parkin and various E3 members, as indicated. The blots above were stripped and reprobed with anti-actin antibody to reflect loading variations. These experiments were repeated 3 times with similar results.

To test our hypothesis that parkin-mediated K63 ubiquitination may be enhanced in cells undergoing proteasomal stress, we next examined the immunoreactivity of anti-UbK63 in Triton-X-100-soluble (S) and -insoluble (P) lysates sequentially prepared from parkin-expressing cells in the presence or absence of proteasome inhibition. We detected a modest but significant increase in the levels of K63-linked polyubiquitination specifically in the P fraction in untreated cells expressing parkin compared to control cells (Figure 1A). Importantly, when these parkin-expressing cells were treated with the proteasome inhibitor, MG132, we observed a dramatic increase in the level of anti-UbK63 immunoreactivity, which again resides predominantly in the P fraction (Figure 1A). The same phenomenon is observed when parkin-expressing cells were treated with PSI and lactacystin, two other proteasome inhibitors but not with DMSO vehicle (Figure S2A-B). Substituting parkin with a truncation mutant deleted of its C-terminal catalytic RING domain (ΔRING) significantly reduces the level of K63 polyubiquitinated proteins in cells treated with MG132, as are substitutions with disease-associated RING mutants, T240R, T415N and G430D (Figure 1B). On the other hand, a parkin mutant carrying the M192L mutation, which resides outside the RING catalytic domain, retains the ability to promote K63-linked polyubiquitination (Figure 1B). Our results thus suggest that proteasome inhibition promotes parkin-mediated K63-linked ubiquitination, an activity that is clearly dependent on the integrity of its RING domain. To extend this finding, we also repeated our experiment with MG132-treated cells expressing other E3 members. Anti-UbK63 immunoblotting of lysates prepared from these variously transfected cells revealed that Siah-1, for which no association with K63-linked polyubiquitination has been reported to date, as well as two other RING-containing E3s, HHARI and Cbl, failed to enhance the levels of K63 polyubiquitinated proteins in the P fraction in response to proteasome inhibition (Figure 1C). However, CHIP (which has been associated with K63-linked ubiquitination) appears capable of mediating the phenomenon (Figure 1C). Taken together, our results demonstrate that in the presence of proteasome inhibition, the K63-linked ubiquitination activity of parkin is significantly enhanced, which results in the accumulation of proteins in detergent-insoluble fractions.

### Proteasome inhibition promotes the recruitment of Ubc13 by parkin

**Figure 2:**
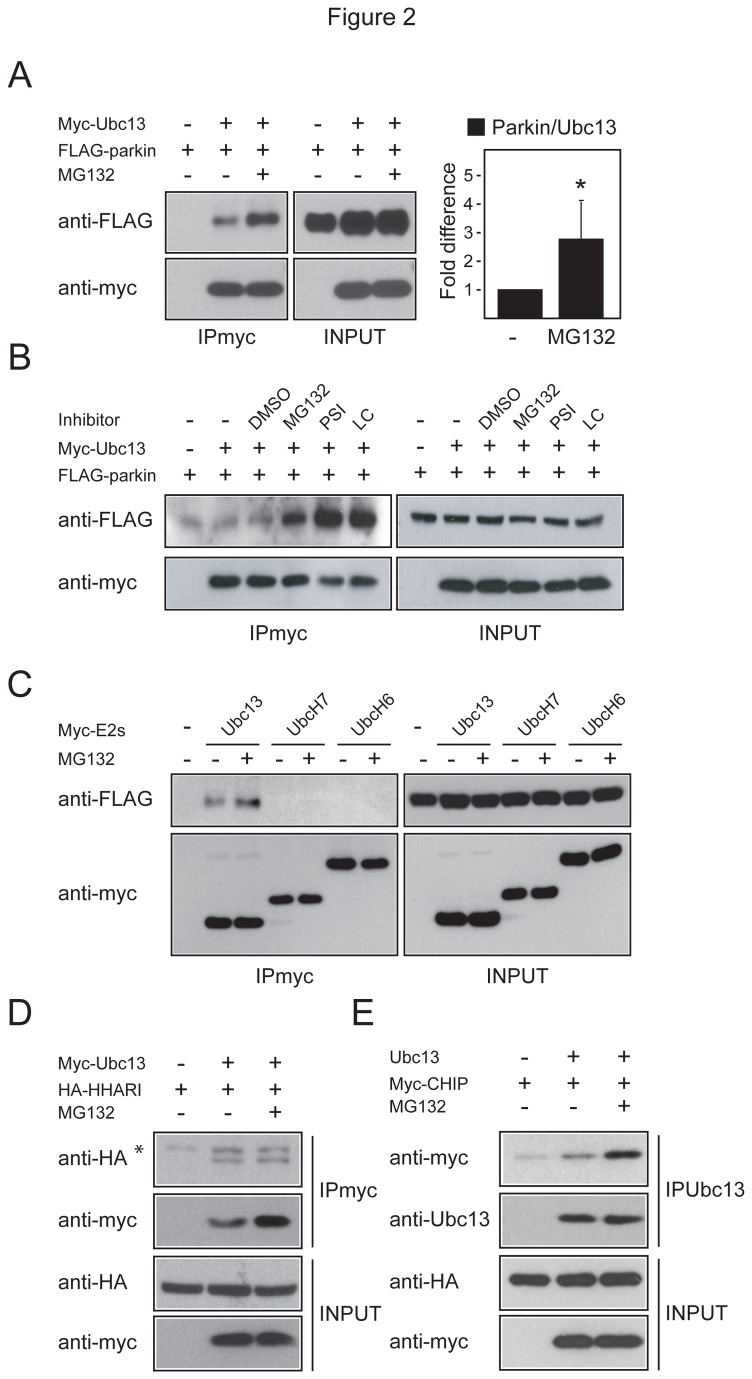
Proteasome inhibition promotes the interaction between parkin and Ubc13. (A) *Left*, A portion of Triton-X-soluble lysates prepared from untreated or MG132-treated HEK293 cells expressing FLAG tagged parkin alone or with myc-tagged Ubc13 were subjected to anti-myc immunoprecipitation followed by anti-FLAG and anti-myc immunoblotting (*IPmyc*). The remainder lysates prepared from these variously transfected cells (*INPUT*) were subjected to anti-FLAG and anti-myc immunoblotting to show the expression levels of FLAG-parkin and myc-Ubc13 respectively. *Right*, Bar graph showing the densitometric levels of parkin that co-immunoprecipitated with Ubc13 from untreated or MG132-treated transfected cells (*P < 0.05). (B-C) Same as above except that experiment included (B) DMSO, PSI and Lactacystin (LC)-treated cells, or (C) myc-tagged UbcH7 or H6 as controls. (D) Same as (A) except that FLAG-tagged parkin was substituted with HA-tagged HHARI and anti-FLAG immunoblotting was replaced by anti-HA immunoblotting. Asterisk denotes non-specific bands. (E) Same as (A) except that FLAG-tagged parkin was substituted with myc-tagged CHIP and anti-FLAG immunoblotting was replaced by anti-myc immunoblotting. Immunoprecipitation was carried out with anti-Ubc13. These experiments were replicated at least three times.

Given that Ubc13 is uniquely associated with K63-linked ubiquitination, and our observation above that parkin-mediated K63-linked ubiquitination is promoted by proteasome inhibition, we surmised that the binding affinity between Ubc13 and parkin may be influenced by the functional status of the proteasome. To address this, we carried out co-immunoprecipation experiments with cells transfected with myc-tagged Ubc13 and FLAG-tagged parkin in the presence or absence of MG132 treatment. As suspected, the amount of parkin that co-immunoprecipitated with Ubc13 is significantly enhanced in the presence of proteasome inhibition (Figures 2A & S2C). Notably, the levels of parkin and Ubc13 are not appreciably affected in MG132-treated cells (Figure 2A), suggesting that their enhanced interaction is likely a result of increased binding affinity for each other. We also observed the same phenomenon with transfected cells treated with two other proteasome inhibitors, PSI and lactacystin, but not with DMSO vehicle (Figure 2B), which correlates with our earlier observation (Figure S2A-B). Further, this phenomenon appears specific to Ubc13 as neither UbcH6 nor H7 mediates such an outcome (Figure 2C). Moreover, unlike parkin, the closely-related E3 member HA-HHARI fails to exhibit increased association with Ubc13 in the presence of proteasome inhibition (Figure 2D), although CHIP1, which enhances K63-linked ubiquitination in the presence of MG132 (Figure 1C), also display increased interaction with Ubc13 under such conditions (Figure 2E). Thus, parkin-Ubc13 interaction appears to be rather specifically enhanced under conditions of proteasome impairment, which provides an explanation for the observed enhancement in K63-linked ubiquitination in MG132-treated parkin-expressing cells. Consistent with this, shRNA-mediated silencing of Ubc13 expression results in a significant reduction in the level of K63-linked ubiquitination in parkin-transfected cells in the presence of MG132 (Figure S2D). Interestingly, we also observed a correlative decrease in parkin expression upon Ubc13 expression silencing, which suggest a role for Ubc13 in stabilizing parkin. Supporting this, we found that the level of parkin is significantly reduced in FLAG-parkin transduced Ubc13-/- MEFs compared to those expressing in wild type MEFs, even in the presence of MG132 treatment (Figure S2E). Notably, MEFs derived from parkin null mice also exhibit a reduction in the level of K63-linked ubiquitination relative to their wild type counterparts, albeit more modestly so, when treated with MG132 (Figure S2F).

**Figure 3:**
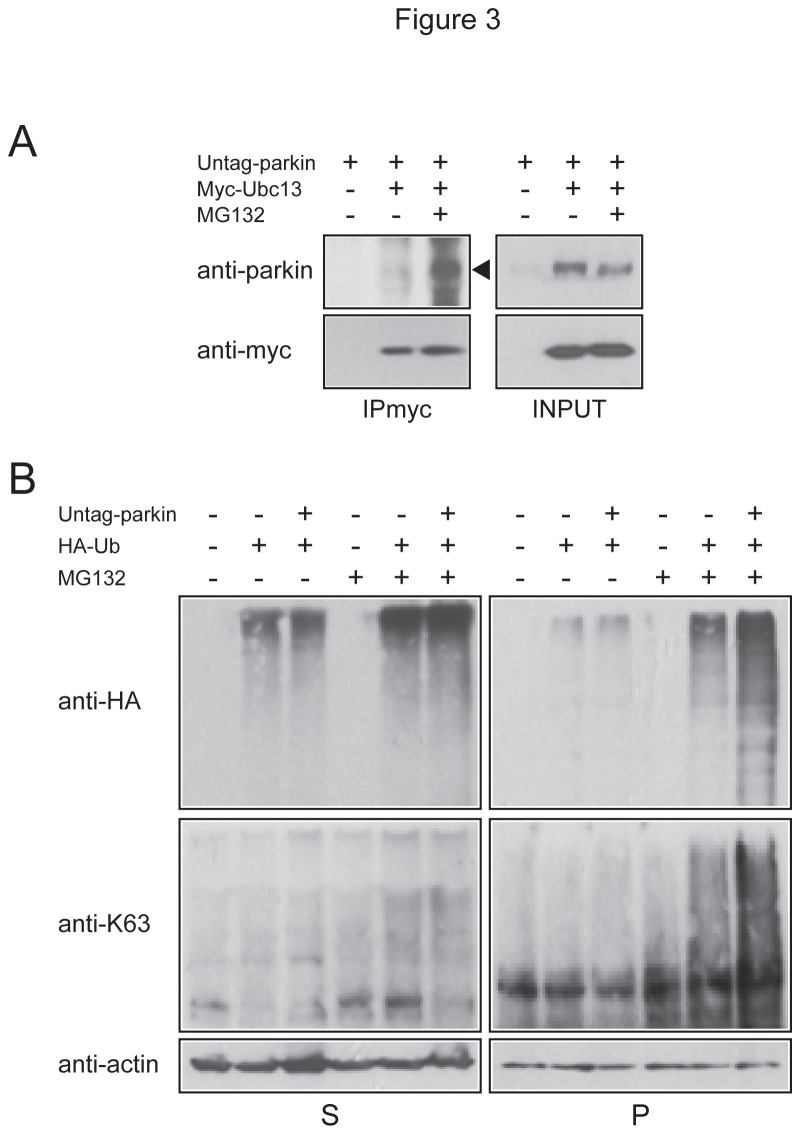
Proteasome inhibition promotes the interaction between untagged parkin and Ubc13 and concomitantly enhances K63-linked ubiquitination. (A) A portion of Triton-X-soluble lysates prepared from untreated or MG132-treated HEK293 cells expressing untagged parkin alone or with myc-tagged Ubc13 were subjected to anti-myc immunoprecipitation followed by anti-parkin and anti-myc immunoblotting (IPmyc). The remainder lysates prepared from these variously transfected cells (INPUT) were also subjected to anti-parkin and anti-myc immunoblotting to show the expression levels of parkin and myc-Ubc13 respectively. (B) Representative anti-HA and anti-K63 ubiquitin immunoblots of cell extracts sequentially prepared with Triton-X 100 (S) and SDS (P)-containing buffer from untreated or MG132-treated HEK cells transfected with HA-tagged ubiquitin, and vector or untagged parkin, as indicated.

In view of the recent demonstration by Chaugule and colleagues that parkin activity is normally repressed by its ubiquitin-like (Ubl) domain (which can be disrupted by N-terminal epitope tagging) [23], we examined whether proteasome inhibition can relieve the repression to promote the interaction between untagged full length parkin and Ubc13 and concomitantly enhance K63-linked ubiquitination. Interestingly, we found that untagged parkin also exhibits a greater affinity for Ubc13 under conditions of proteasome inhibition (Figure 3A) and that the levels of K63 polyubiquitin-modified proteins are specifically enhanced in the pellet fraction in untagged parkin-transfected cells in the presence of MG132 treatment (Figure 3B). Thus, proteasome inhibition results in an increased association between Ubc13 and FLAG-tagged or untagged parkin species, both events leading to enhanced K63-linked ubiquitination.

Notwithstanding the above, how proteasome inhibition increases the affinity between parkin and Ubc13 remains unclear, although a recent study by Sha and colleagues have demonstrated that parkin phosphorylation by PINK1 promotes its interaction with Ubc13/Uev1a and concomitantly activates its K63-linked ubiquitination activity [24]. We therefore wondered whether parkin is similarly phosphorylated under conditions of proteasome impairment. To address this, we immunoprecipitated parkin from transfected cells in the presence or absence of MG132 and examined its phosphorylation status by immunoblotting with anti-phosphoserine and anti-phosphothreonine antibodies [similar to the ones described by Sha et al [24]]. However, we failed to detect any evidence of parkin phosphorylation in the absence or presence of proteasome inhibition, or even in the presence of PINK1 co-expression, which we have included as a control (Figure S3A & B). As the detection of protein serine/threonine phosphorylation using phospho-specific antibodies is well known to be tricky, we also analyzed the electrophoretic mobility of parkin prepared from untreated or MG132-treated cells but found no alteration to suggest that parkin is phosphorylated in the presence of proteasome inhibition (Figure S3C). Thus, parkin phosphorylation does not appear to be responsible for its increased affinity for Ubc13 under conditions of proteasomal stress.

### Synphilin-1 and mutant DJ1 over-expression further promotes parkin-Ubc13 interaction

**Figure 4:**
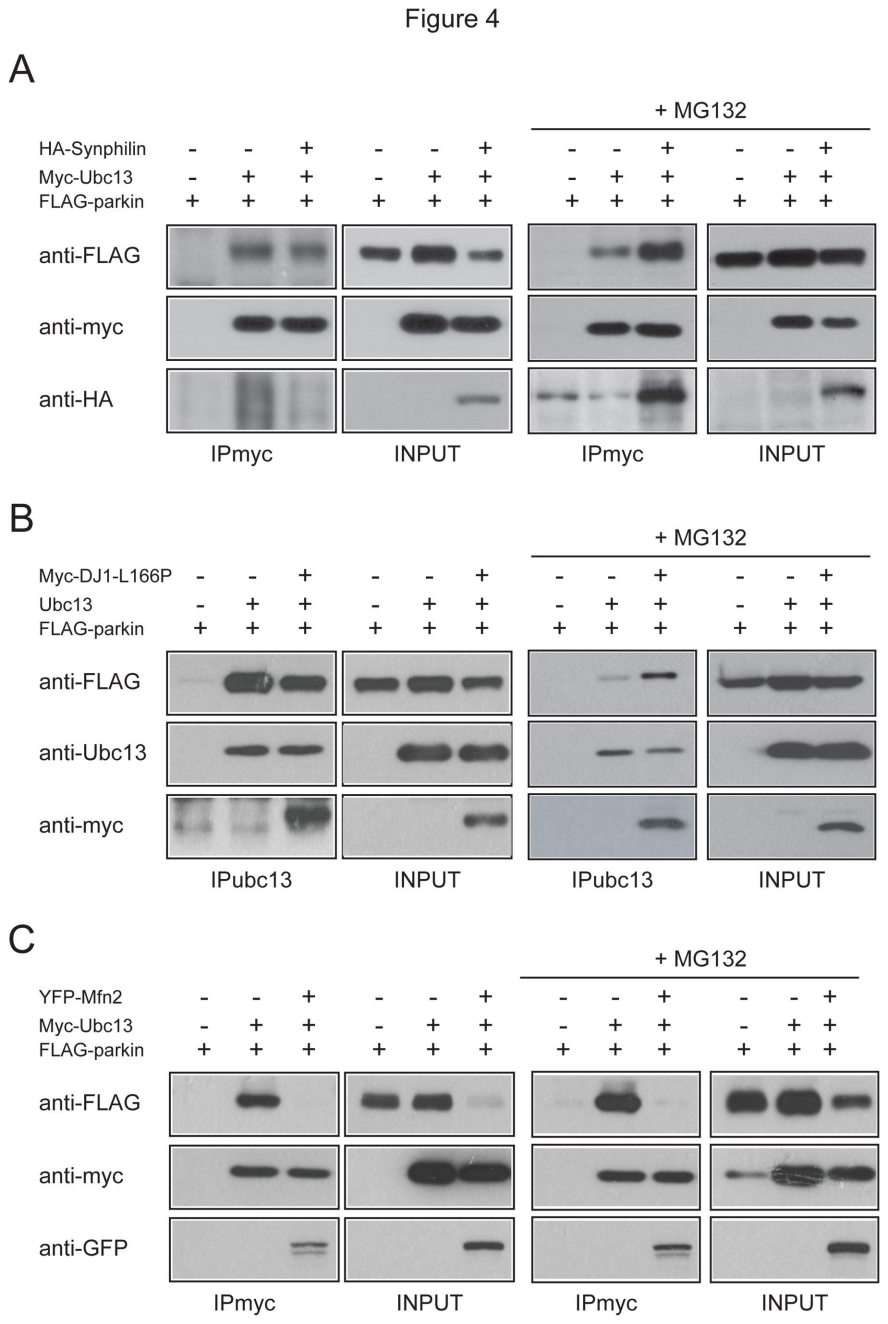
Effects of substrate overloading on parkin-Ubc13 association. (A) A portion of Triton-X-soluble lysates prepared from untreated and MG132-treated HEK293 cells expressing FLAG-tagged parkin alone or with myc-tagged Ubc13 and/or HA-synphilin were subjected to anti-myc immunoprecipitation followed by anti-FLAG, anti-myc and anti-HA immunoblotting (IPmyc). The expression levels of the transfected cDNAs are shown in INPUT blots. (B) As in (A) except that HA-synphilin is replaced by myc-DJ1 mutant and that immunoprecipitation was carried out using anti-Ubc13 antibody. (C) As in (A) except that HA-synphilin is replaced by YFP-Mfn2.

Previously, we have demonstrated that synphilin-1 ubiquitination by parkin and Siah-1 occurs via K63 and K48 respectively [11]. Consistent with this, and with our results above, we found that parkin, but not Siah-1 or several other related E3 members, promotes synphilin-1 accumulation in the Triton-X-insoluble P fraction (Figure S4A & B). Not surprisingly, this phenomenon could be mimicked by co-expressing synphilin-1 with K63 ubiquitin mutant, although the lysineless K0 mutant also triggers similar outcome (Figure S4C & D). Given the recent finding by Winklhofer’s group that parkin is capable of mediating linear ubiquitin chain assembly [25], there is a possibility that the K0 mutant could support linear ubiquitination of synphilin-1 in the presence of parkin thereby leading to the stabilization of the protein. As linear and K63 ubiquitin chains are structurally quite similar, we tested to see if the K63 antibody might recognize K0-ubiquitinated proteins but found no evidence of their cross-reactivity (Figure S5). Notwithstanding this, given that synphilin-1 is ubiquitinated by parkin via K63, we reasoned that synphilin-1 over-expression in cells treated with MG132 might further promote the recruitment of Ubc13 by parkin. Indeed, we found that parkin-Ubc13 interaction occurs significantly more strongly in the presence of synphilin-1 over-expression (Figure 4A). This phenomenon is however dependent on proteasome inhibition as the enhancement of parkin and Ubc13 interaction in the presence of synphilin-1 did not take place in untreated transfected cells (Figure 4A). Next, we examined parkin-Ubc13 interaction in MG132-treated cells over-expressing DJ-1 L166P mutant. Based on studies involving ubiquitin mutant over-expression, Olzmann et al has shown that under conditions of proteasome impairment, parkin-mediated ubiquitination of DJ-1 L166P mutant also occurs via K63 [16]. Similar to synphilin-1, we found that over-expression of DJ-1 L166P in cells significantly enhanced the binding between Ubc13 and parkin in the presence but not absence of MG132 (Figure 4B). Our results thus suggest that under conditions of proteasome impairment, parkin preferentially recruits Ubc13 to mediate K63-linked ubiquitination on selected substrates. To support this further, we repeated our experiments with mitofusin 2 (Mfn2), a substrate of parkin whose degradation is accelerated in the presence of the E3 [26], suggesting that parkin-mediated ubiquitination of Mfn2 is unlikely to be K63-linked. In this case, we failed to observe any enhancement in parkin-Ubc13 interaction in the absence or presence of proteasome inhibition (Figure 4C). However, a trivial explanation for this is that the availability of parkin is dramatically reduced in the presence of Mfn2, which is rather curious. Indeed, Mfn2 over-expression appears to promote parkin degradation in our hands, which is mitigated in the presence of MG132 (Figure 4C). Although we remain intrigued by this observation, it is clear that the added enhancement of parkin-Ubc13 interaction that we have observed with synphilin-1 and DJ1L166P in the presence of proteasome inhibition does not apply to all parkin substrates.

### Parkin facilitates the autophagic clearance of synphilin-1 inclusions formed in the presence of proteasome inhibition

**Figure 5:**
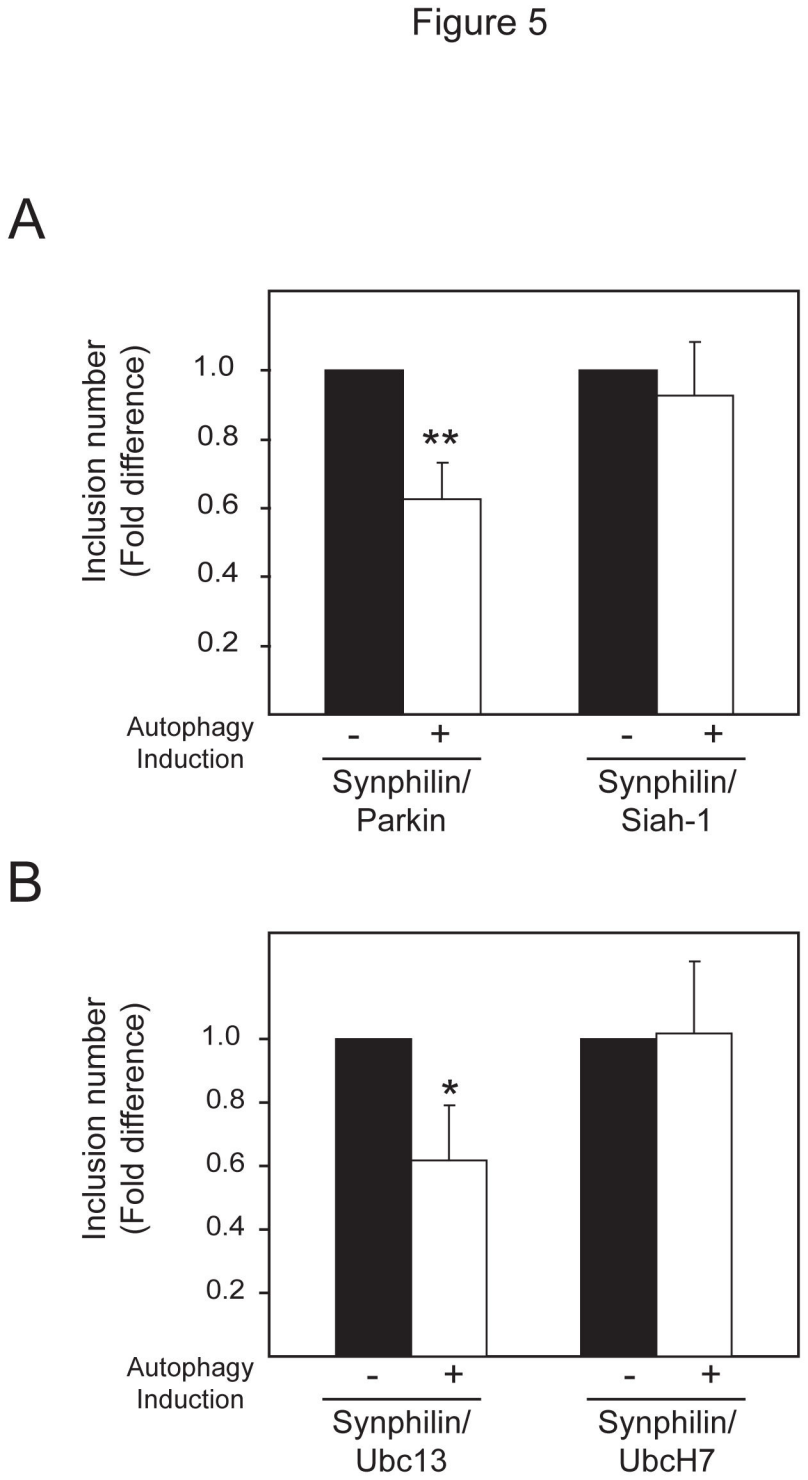
Autophagy clearance of synphilin-1-positive inclusions is enhanced in the presence of parkin or Ubc13 overexpression. (A) Bar graph showing the relative fold difference in the number of synphilin-1-positive inclusions formed in lactcystin-treated cells co-expressing (a) parkin or Siah-1, or (b) Ubc13/Uev1a or UbcH7, that were allowed to recover in normal (no autophagy induction, -) or low serum (autophagy induced, +) medium (*P < 0.05, **P < 0.001 *vs* control group).

Given the recent finding by our group and others that K63 polyubiquitin may act as a signal to target proteins to the aggresome-autophagy pathway [6,16], it is tempting to speculate that the autophagic clearance of synphilin-1 inclusions formed under conditions of proteasome inhibition may be facilitated by parkin. By means of an inclusion clearance assay described previously [6], we found that exogenously-introduced parkin, but not Siah-1, promotes the autophagic clearance of synphilin-1 inclusions generated in cells treated with the proteasome inhibitor, lactacystin (Figure 5A). Corroborating with this, we further found that whereas synphilin-1 inclusions generated in cells co-expressing Ubc13 and Uev1a are amenable to clearance by autophagy, those formed in cells expressing UbcH7 are apparently resistant to autophagy-mediated clearance (Figure 5B). Thus, the enhanced recruitment of Ubc13 by parkin in the presence of synphilin-1 over-expression and proteasome inhibition appears to be a cellular response in favour of alternate clearance of this parkin substrate via the autophagy route.

### K63-polyubiquitination protects cells against proteasome inhibition-induced cytotoxicity

**Figure 6:**
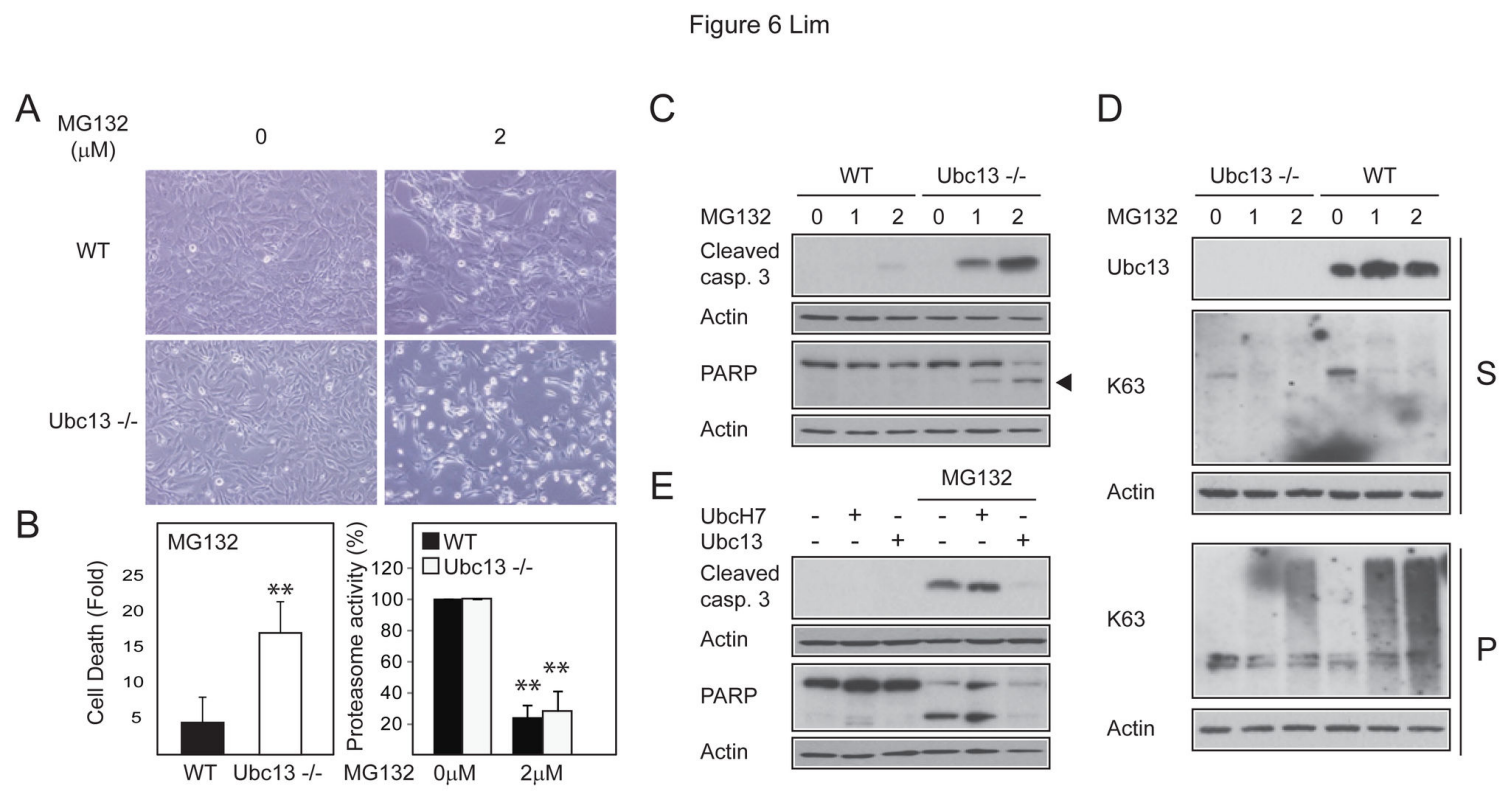
Ubc13 null MEFs are vulnerable to proteasome inhibition-induced cytotoxicity. (A) Representative phase-contrast images showing an obvious increase in the population of rounded, refractile cells in MG132-treated Ubc13 -/- MEFs relative to their untreated or MG132-treated wild type counterparts. (B) The cell viability (left) (Fold difference between MG132-treated vs. untreated cells) and proteasome activity (right) of wild type and Ubc13 -/- in the absence or presence of MG132 treatment (16h) were measured and plotted as a bar graph respectively. (C) Immunoblots showing the levels of cleaved caspase 3 and cleaved PARP in lysates prepared from wild type and Ubc13 -/- MEFs in the absence or presence of MG132 treatment (1 or 2 µM, as indicated). (D) Same as (C) as anti-K63 antibodies were used. (E) Immunoblots showing the levels of cleaved caspase 3 and cleaved PARP in lysates prepared from Ubc13 -/- MEFs transduced with lenti-Ubc13 or UbcH7, as indicated, in the absence or presence of 2 µM MG132.

We surmised that the upregulation of K63-linked ubiquitination in the presence of proteasome inhibition may be a cellular protective response. To address this, we subjected wild type and Ubc13-/- MEFs to MG132 treatment to examine their relative susceptibility to proteasome inhibition-induced cell death. As per our speculated protective role of K63-linked ubiquitination, we observed that a significant population of the MG132-treated Ubc13-/- MEFs became rounded and reflective, i.e. indicative of dying cells whereas wild type MEFs treated with MG132 were relatively spared of these features (Figure 6A). Quantitative measurement of cell death at this time point by means of flow cytometry correlates well with our morphological observations, which we further showed is related to proteasome inhibition (Figure 6B). Moreover, immunoblotting of lysates prepared from these cells revealed a dose-dependent enhancement in the levels of two cell death markers, i.e. cleaved caspase 3 and cleaved PARP in Ubc13-/- MEFs in the presence of MG132-mediated proteasome inhibition relative to their untreated or wild type MEF counterparts (Figure 6C). Together, these results suggest that the inability of Ubc13-/- MEFs to promote K63-linked ubiquitination under conditions of proteasome impairment renders them vulnerable to cell death. Consistent with this, we detected significantly reduced levels (although curiously not complete absence) of K63-linked ubiquitination in Ubc13-/- MEFs in the presence of MG132 treatment, the levels of which comparatively increased robustly in a dose-dependent manner in wild type MEFs in response to MG132-mediated proteasome inhibition (Figure 6D). Importantly, we further demonstrated that MG132-induced cell death in Ubc13-/- MEFs can be rescued by the ectopic expression of exogenous Ubc13, as evident by the reduced levels of cleaved caspase 3 and cleaved PARP in these cells compared to un-transduced Ubc13-/- MEFs (Figure 6E). Conversely, the introduction of UbcH7 into Ubc13-/- MEFs appears to aggravate the extent of MG132-induced cell death in these cells instead of rescuing them (Figure 6E). Thus, the upregulation of Ubc13-mediated K63-linked ubiquitination under conditions of proteasome impairment appears to fulfil a protective role.

## Discussion

In essence, the main finding of our current study is that proteasome inhibition promotes parkin-Ubc13 interaction and concomitantly enhances parkin-mediated K63-linked ubiqiuitination. Our study thus identifies a mechanism by which parkin could promote K63-linked ubiquitin modification in cells undergoing proteolytic stress, which appears to facilitate the subsequent clearance of selected parkin substrates via autophagy. How parkin is modified such that its affinity for Ubc13 is increased in the presence of proteasome inhibition however remains elusive. Nonetheless, our study suggests a role for parkin-mediated K63 ubiquitination in maintaining cellular protein homeostasis, especially during periods when the proteasome is heavily burdened or impaired.

*Parkin* was originally identified as a gene whose mutations are causative of autosomal recessive parkinsonism [8]. . The disease is characterized by an earlier onset of symptoms, typically before the age of 40 years, suggesting that lack of functional parkin markedly accelerates the degeneration process, and thereby the role of parkin as a key protector against neuronal death. Indeed, parkin can afford considerable protection against a remarkably wide spectrum of cellular stress [27,28], including those that promote proteasome dysfunction [29–31]. However, the mechanism underlying the preservation of proteasome function by parkin is hitherto unclear, although we have originally proposed that parkin-mediated K63-linked ubiquitination may serve to mitigate proteasome overloading by diverting the substrate load away from the machinery [4]. Notably, we have previously demonstrated that parkin promotes, via its K63-linked ubiquitination activity, the formation of synphilin-1 inclusions [11]. Consistent with this, we showed here that parkin selectively enhances the accumulation of synphilin-1 in the pellet fraction of cell lysates expressing the two proteins. Moreover, in the absence of exogenously-introduced substrates, parkin apparently also leads to a significant global enhancement of K63-linked ubiquitinated proteins especially in the presence of proteasome inhibition. Thus, parkin appears to be one of the key cellular mediators of K63-linked ubiquitination in times of proteolytic stress, although other E3 ligases such as CHIP that are capable of K63 ubiquitin chain assembly would presumably play a collaborative role with parkin to enhance this form of ubiquitination under such conditions. Consistent with this, although we detected a reduction in the level of K63-linked ubiquitination in MG132-treated parkin null fibroblasts relative to their wild type counterparts (Figure S2F), we consider the decrease to be modest and are open to the scenario that other E3 ligases competent in mediating K63-linked ubiquitination may also be involved.

Mechanistically, how proteasome inhibition promotes the affinity between parkin and Ubc13 is currently unclear to us. Although parkin phosphorylation by PINK1 was previously demonstrated by Sha et al to promote its interaction with Ubc13/Uev1a [24], we found no evidence that this post-translational modification is responsible for the enhanced parkin-Ubc13 association that we have observed in the context of proteasome impairment. Interestingly, proteasome inhibition also leads to increased binding of Ubc13 with untagged parkin, which was proposed by Chaugule et al to exist in an auto-inhibited state by virtue of the interaction between the N-terminal Ubl domain of parkin and a region close to its catalytic RING2 (i.e. which results in the formation of a closed loop) [23]. It is noteworthy to highlight that we have previously also proposed a similar model of parkin activity repression, although we found that the unique region of parkin (i.e. between Ubl and RING1) serves as the inhibitory domain [32]. Recently, several groups have reported the crystal structure of parkin that supports our model of parkin catalytic inhibition [33–35]. Collectively, these groups found that parkin is normally kept in auto-inhibited state by two key mechanisms – (i) a linker region between IBR and RING2 that is positioned in a configuration that would block the conserved E2 ~ Ub binding site of RING1 (thus denying access to E2), and (ii) an interface that forms between the unique region of parkin and RING2 that buries the catalytic site of RING2. Given this, it is attractive to speculate that proteasome inhibition may promote a conformational change in parkin structure (through some unknown mechanism) that enhances its selective recruitment of Ubc13, leading to increased K63-linked ubiquitination.

Interestingly, parkin-related cases are frequently (although not absolutely) devoid of Lewy bodies (LB), the classic histological hallmark of Parkinson’s disease (PD), suggesting that the catalytic activity of parkin may play a role in LB biogenesis [36]. We found here that disease-associated RING mutants of parkin, including T240R, T415N and G430D, fail to promote cellular K63-linked ubiquitination in the presence of proteasome inhibition. Notably, parkin-mediated K63-linked ubiquitination is apparently also important for the activation of the pro-survival NFκB signaling in times of moderate cellular stress [37]. Related to this, a recent report demonstrated that parkin is capable of collaborating with LUBAC to mediate linear ubiquitin chain assembly, that is important to prevent mitochondrial impairment under cellular stress [25]. Although we cannot exclude the participation of parkin-mediated linear chains in our observations, our studies in Ubc13 knockout cells would support a pro-survival role for K63-linked ubiquitination in times of proteolytic stress. Parkin mutants that are incapable of this mode of ubiquitin modification would therefore be expected to put cells at greater risk of degeneration in times of stress.

Given that K63-linked polyubiquitination of proteins is generally (although not obligatory) uncoupled from the proteasome, it is conceivable that enhanced cellular ubiquitin modification of proteins via K63 would promote their accumulation and subsequent aggregation in the cell. Indeed, we have demonstrated previously with ubiquitin mutant over-expression [6,11] and in the present study with K63-specific antibody that this is the case, i.e. K63 ubiquitinated proteins tends to accumulate and specifically in detergent-insoluble fractions of cell lysate (Figure S1). Corroborating with our studies, Olzmann et al have previously identified parkin-mediated K63-linked ubiquitination as a signal that couples misfolded DJ-1 to the dynein complex via HDAC6 and thereby promoting the sequestration of proteins into aggresomes [16]. Since aggresomes are commonly thought to act as staging grounds for the disposal of protein aggregates *via* the autophagic route, their result suggests that parkin may facilitate the clearance of proteins by autophagy. We have subsequently extended the study by Olzmann et al by showing that K63-linked polyubiquitin acts as a novel cargo selection signal for the autophagy apparatus [5,6]. Further, we demonstrated in the present study that parkin-mediated ubiquitination of synphilin-1, which we have shown previously to occur via K63 [11], facilitates its clearance by autophagy. Moreover, the same phenomenon could be observed when synphilin-1 is co-expressed with Ubc13/Uev1a but not UbcH7. It is however noteworthy that the degradation of both synphilin-1 and DJ1-L166P could occur via the proteasome under normal conditions. In the case of synphilin-1, Siah-1 and -2, as well as Dorfin, have been identified to be its degradation-associated E3s [38–40]. Thus, proteins like synphilin-1 and DJ1-L166P, which could be modified by K48- or K63-linked ubiquitin, appear to be under dynamic cellular control. Collectively, these studies suggest that enhanced parkin-mediated K63-linked ubiquitination (by virtue of its increased interaction with Ubc13) may help to divert selected cargo proteins, like synphilin-1 and DJ1-L166P, away from the proteasome in times of overloading to enable their subsequent clearance by the autophagy system. Consistent with the protective role of K63-linked ubiquitination under conditions of proteasome impairment, we demonstrated that Ubc13 -/- MEFs that are presumably incapable of mediating this mode of ubiquitin modification are significantly more susceptible compared to wild type MEFs to the toxicity induced by MG132, a defect that can be rescued by exogenous introduction of Ubc13 but not UbcH7 (which is not associated with K63 polyubiquitination).

Notably, a recent study by Paine et al that involved the use of linkage-specific antibody demonstrated that K63-linked ubiquitin pathology accompanies proteasome impairment in a mouse model of proteasome dysfunction [41]. However, although the same study also revealed the presence of K63-linked ubiquitin in post-mortem PD brains, the immunoreactivity is only found in a small percentage (~5%) of LBs examined. On the other hand, marinesco bodies appear well stained. Further, a previous MS-based analysis of α-synuclein ubiquitination in purified LB by another group revealed the presence of K48-linked ubiquitin but not K63-linked ubiquitin [42]. These related studies by others are therefore seemingly at odds with ours, which imply a role for parkin-mediated K63-linked ubiquitination in LB formation. A plausible explanation that could account for the discrepancy, aside from the poor sensitivity of current linkage-specific antibodies for endogenous ubiquitin, is that the ubiquitin tag is not static but a dynamic protein modification, i.e. it is conceivable that polyubiquitin chains including K63-linked chains are progressively modified by deubiquitinating enzymes with time, which could present a confounding factor. Lending support to this, Wang and colleagues [43] recently demonstrated that the formation of aggresomes mediated by mutant superoxide dismutase 1 (SOD1) is dependent on ataxin 3-catalyzed editing of K63-linked polyubiquitin chain on SOD1 (presumably to a correct length). Conversely, knockdown of ataxin-3 decreases mutant SOD1 aggresome formation and increases cell death induced by mutant SOD1. Thus, the chain length of K63 polyubiquitin-modified proteins is under dynamic regulation. Alternatively, since neurons are capable of constitutive autophagy, we have recently proposed that the presence of LB may reflect a failure by autophagy to remove the precursors of these structures [44]. This could obviously arise from gross autophagy system dysfunction, or alternately, from an inability of certain types of LB to recruit the autophagy apparatus efficiently, perhaps because (for some unknown reasons) they lacked the K63-linked ubiquitin tag, amongst other important autophagy recruitment components. Relevant to this, we have previously demonstrated that the composition of an aggresome influences its clearance by autophagy [45]. Notwithstanding the unresolved issues, our present study potentially offers a mechanistic explanation as to why parkin could afford considerable protection against proteasome dysfunction elicited by various endogenous or exogenous insults [29–31]. A role for parkin in the triage of proteins between proteasomal and lysosomal degradation thus appears attractive to us, although precisely how proteasome inhibition promotes the recruitment of Ubc13 by parkin awaits further clarifications.

## Supporting Information

Figure S1 **K63 polyubiquitinated proteins reside in detergent-insoluble fractions of cell lysates.** (A) Representative anti-ubiquitin (FK1), anti-K48 or -K63 immunoblots of chemically synthesized K48 or K63 polyubiquitin chains (BIOMOL), as indicated. (B) Representative anti-HA and anti-K63 immunoblots of cell extracts sequentially prepared with Triton-X 100 (S) and SDS (P)-containing buffer from HEK cells transfected with various ubiquitin species, as indicated. The blots above were stripped and reprobed with anti-actin antibody to reflect loading variations. (C) Representative anti-HA and anti-K63 immunoblots of S and P fractions of HEK cells transfected with HA-tagged wild type ubiquitin and various myc-tagged E2 species, as indicated. Top and bottom arrows point to Uev1a and Ubc13 respectively.
(PDF)

Figure S2 **K63-polyubiquitination is enhanced in parkin-expressing cells in the presence of proteasome inhibition.** (A) Representative anti-K63 and anti-FLAG immunoblots of cell extracts sequentially prepared with Triton-X 100 (S) and SDS (P)-containing buffer from control HEK cells or those transfected with HA-Ubiquitin alone or with FLAG-tagged parkin in the absence or presence of various proteasome inhibitors, as indicated. The blots above were stripped and reprobed with anti-actin antibody to reflect loading variations. These experiments were duplicated with similar results. (B) Bar graph showing the chymotrypsin-like proteasome activities of lysates prepared from untreated cells or those treated with various proteasome inhibitors, as indicated (*P < 0.05, **P < 0.001 vs. column 1, Student’s *t*-test). Control refers to lysates added with MG132 in vitro (C) *Left*, A portion of Triton-X-soluble lysates prepared from untreated or MG132-treated HEK293 cells expressing FLAG tagged parkin alone or with myc-tagged Ubc13 were subjected to anti-myc immunoprecipitation followed by anti-FLAG and anti-myc immunoblotting (*IPmyc*). The remainder lysates prepared from these variously transfected cells (*INPUT*) were subjected to anti-FLAG and anti-myc immunoblotting to show the expression levels of FLAG-parkin and myc-Ubc13 respectively. These experiments were replicated at least three times. (D) Anti-K63 immunoblot of lysates prepared from FLAG-parkin transfected MG132-treated cells in the absence or presence of 2 shRNA species to Ubc13 or control shRNA. The blots above were stripped and reprobed with anti-actin antibody to reflect loading variations. The efficiency of the Ubc13 knockdown is shown in the anti-Ubc13 blot. Notice that the level of parkin as revealed by anti-parkin blot is reduced in the presence of Ubc13 silencing. (E) Anti-parkin immunoblot of lysates prepared from FLAG-parkin transduced WT or Ubc13-/- MEFs in the absence or presence of MG132 treatment, as indicated. The blot was stripped and reprobed with anti-actin antibody to reflect loading variations. (F) Anti-K63 and anti-parkin immunoblot of lysates prepared from wild type (WT) and parkin KO MEFs in the absence or presence of MG132 treatment, as indicated. The blots above were stripped and reprobed with anti-actin antibody to reflect loading variations. These experiments were duplicated.
(PDF)

Figure S3 **Parkin does not appear to be phosphorylated in the presence of MG132.** (A) Anti-phosphoserine and anti-phosphothreonine immunoblots showing the absence of serine/threonine phosphorylation of immunoprecipitated FLAG-tagged parkin in the absence or presence of MG132 treatment or PINK1 co-expression. (B) Anti-phosphoserine and anti-phosphothreonine immunoblots of lysates prepared from cells treated with DMSO or Calyculin a, a potent protein phosphatase inhibitor, shows that the antibodies work fine. (C) Anti-FLAG immunoblotting of 2D gel fractionated cell lysate prepared from FLAG-tagged parkin transfected cells in the absence or presence of MG132 treatment. Note that the parkin-positive spot remains unmodified in both cases (top and middle panels). As a control, when a phospho-mimetic parkin T175D mutant is co-transfected with wild type (WT) parkin, a more acidic parkin-positive spot (arrowhead) can be observed alongside the unmodified one (bottom panel).
(PDF)

Figure S4 **Accumulation of synphilin-1 in cells expressing K63 mutant ubiquitin.** (A) Cell extracts sequentially prepared with Triton-X 100 (S) and SDS (P)-containing buffer from HEK cells transfected with HA-synphilin alone or with FLAG-parkin, myc-Siah-1 or -2, myc-CHIP or GFP dorfin were subjected to immunoblotting with various antibodies, as indicated. Asterisk denotes non-specific bands. Equal loading of the different cell lysates was verified by anti-actin immunoblotting. (B) Bar graphs showing the steady state levels of HA-synphilin in S and P fractions of cell lysate after normalization to their respective loading controls (*P < 0.05, **P < 0.001, Student’s *t*-test). (C–D) Representative anti-myc and anti-HA immunoblots of cell extracts sequentially prepared with Triton-X 100 (S) and SDS (P)-containing buffer from HEK cells transfected with myc-tagged synphilin-1 (Myc-SP) and various ubiquitin species, as indicated. Equal loading of the different cell lysates was verified by anti-actin immunoblotting.
(PDF)

Figure S5 **Anti-K63 antibody does not cross react with K0 ubiquitinated proteins.** Representative anti-myc, anti-K63 and anti-HA immunoblots of cell extracts sequentially prepared with Triton-X 100 (S) and SDS (P)-containing buffer from HEK cells transfected with myc-tagged synphilin-1 (Myc-SP) and K0, K63 or K6 ubiquitin mutant, as indicated. Equal loading of the different cell lysates was verified by anti-actin immunoblotting.
(PDF)
