# Supplementary figures and images for: Proteasome Inhibition Promotes Parkin-Ubc13 Interaction and Lysine 63-Linked Ubiquitination
Source: PLoS One. 2013 Sep 2;8(9):e73235. doi: 10.1371/journal.pone.0073235 (PMC3759450; doi:10.1371/journal.pone.0073235)

Figure S1

A

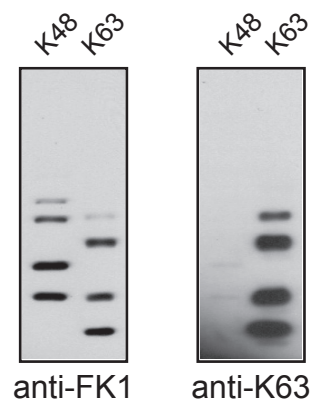

B

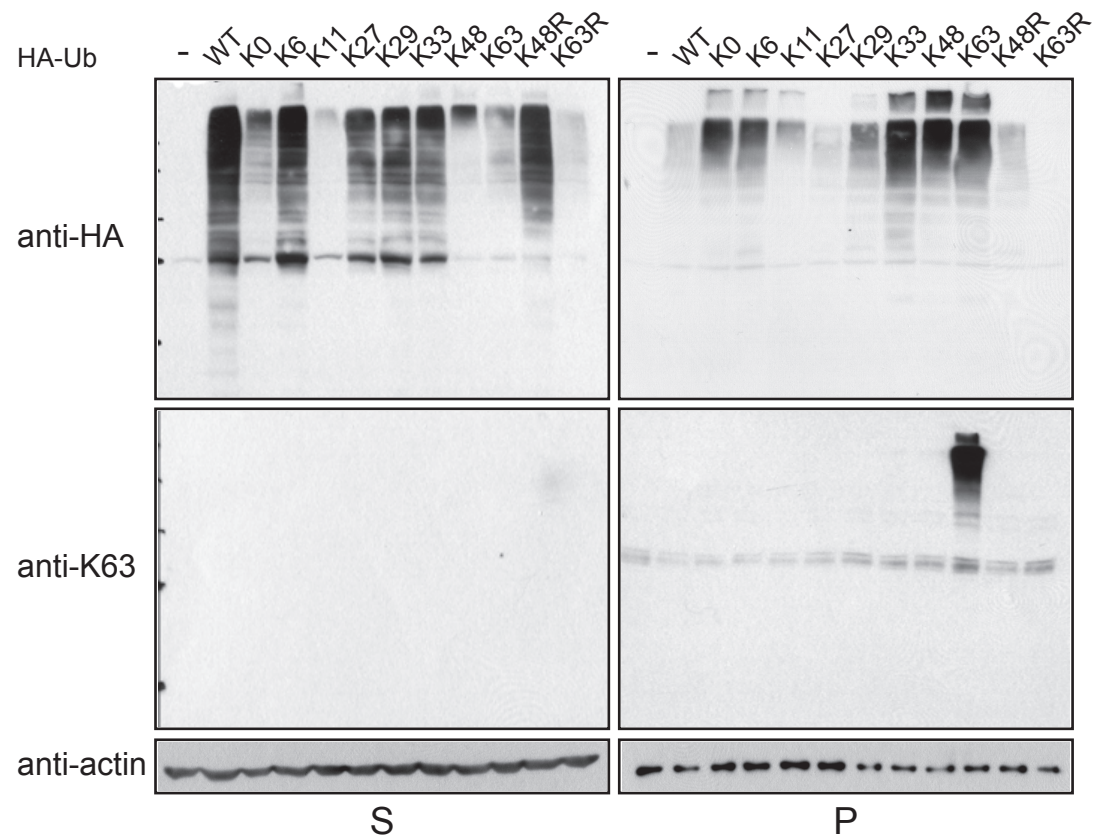

C

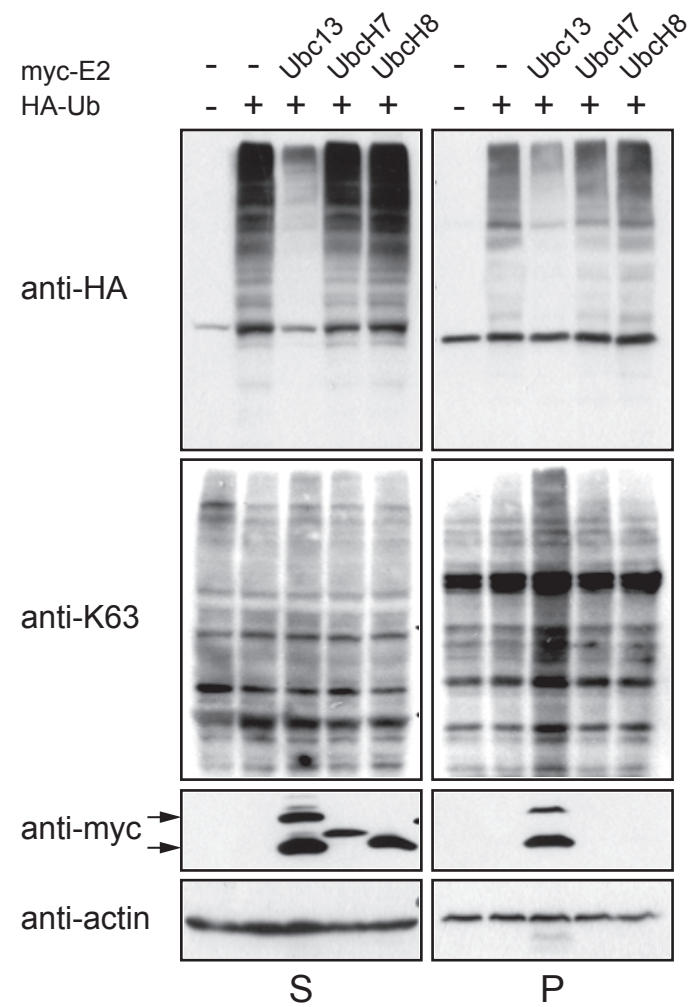

Supplement: Figure S1 — K63 polyubiquitinated proteins reside in detergent-insoluble fractions of cell lysates. (A) Representative anti-ubiquitin (FK1), anti-K48 or -K63 immunoblots of chemically synthesized K48 or K63 polyubiquitin chains (BIOMOL), as indicated. (B) Representative anti-HA and anti-K63 immunoblots of cell extracts sequentially prepared with Triton-X 100 (S) and SDS (P)-containing buffer from HEK cells transfected with various ubiquitin species, as indicated. The blots above were stripped and reprobed with anti-actin antibody to reflect loading variations. (C) Representative anti-HA and anti-K63 immunoblots of S and P fractions of HEK cells transfected with HA-tagged wild type ubiquitin and various myc-tagged E2 species, as indicated. Top and bottom arrows point to Uev1a and Ubc13 respectively. (PDF) [file pone.0073235.s001.pdf]

Figure S2

**A**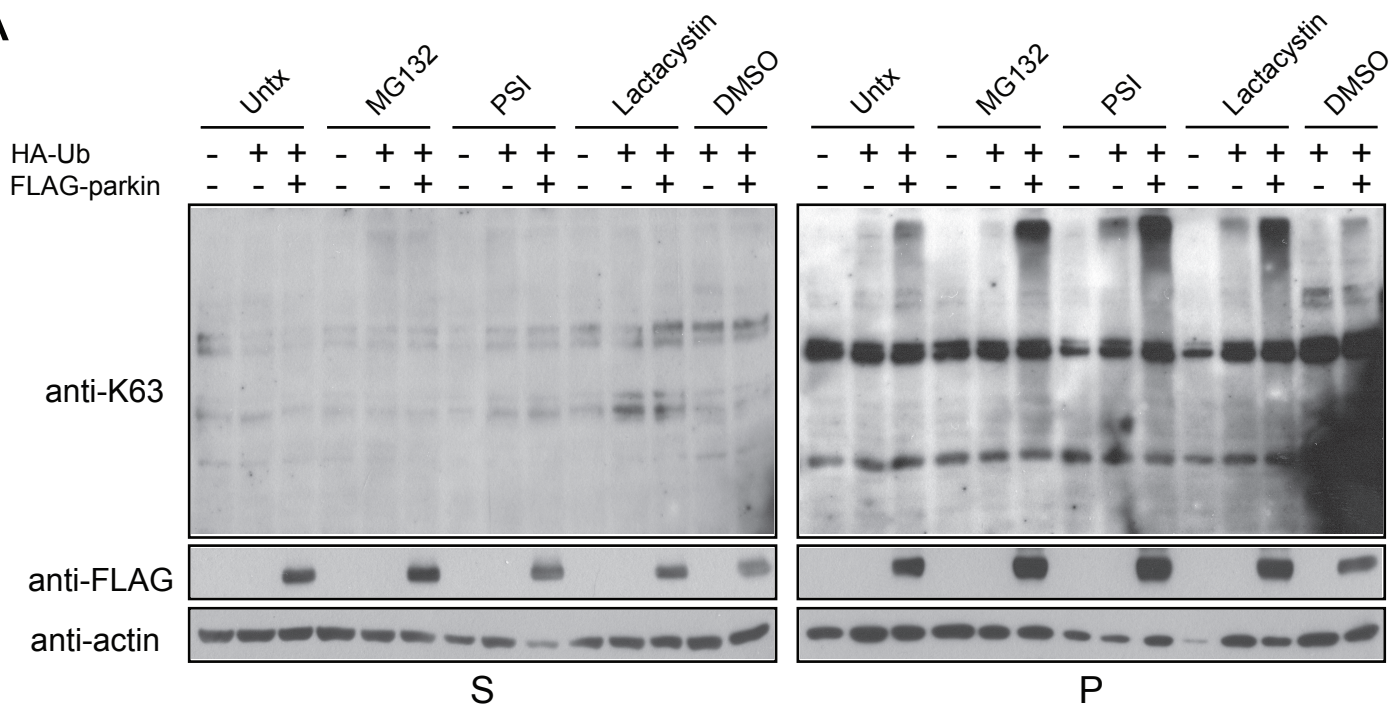**B**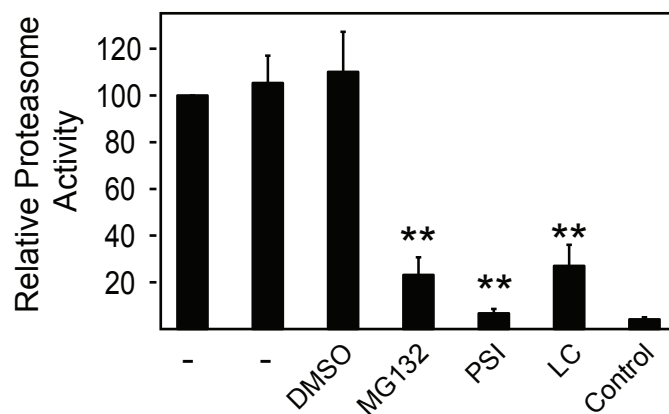**C**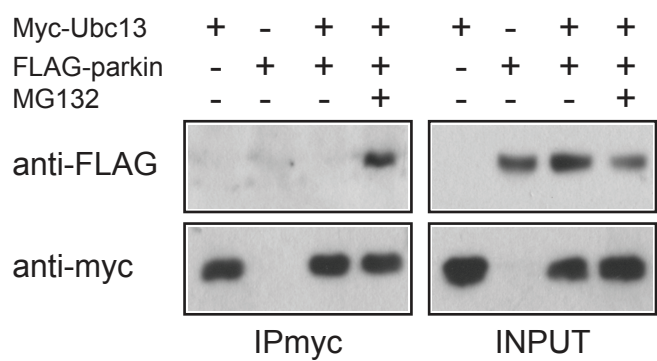**E**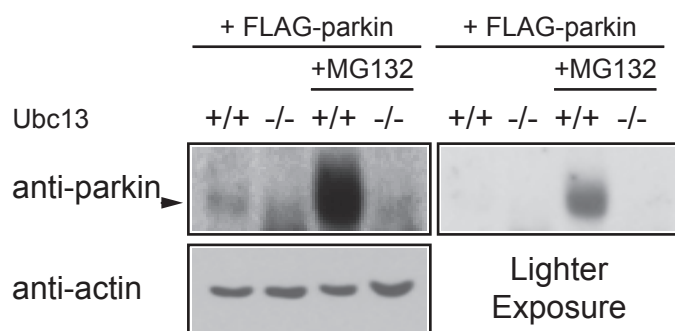**D**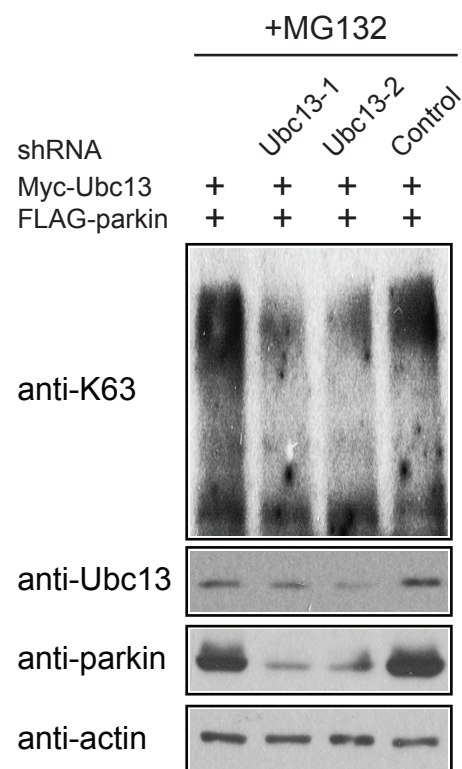**F**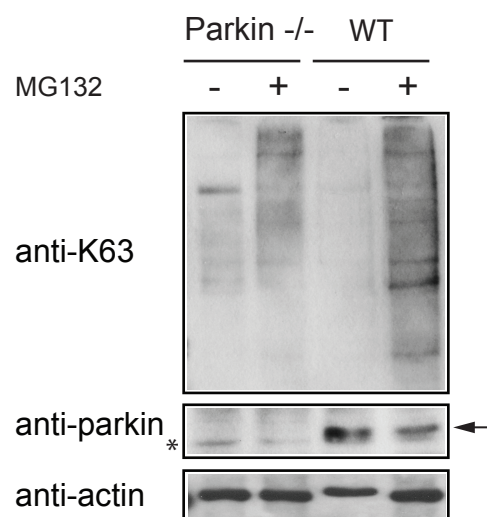

Supplement: Figure S2 — K63-polyubiquitination is enhanced in parkin-expressing cells in the presence of proteasome inhibition. (A) Representative anti-K63 and anti-FLAG immunoblots of cell extracts sequentially prepared with Triton-X 100 (S) and SDS (P)-containing buffer from control HEK cells or those transfected with HA-Ubiquitin alone or with FLAG-tagged parkin in the absence or presence of various proteasome inhibitors, as indicated. The blots above were stripped and reprobed with anti-actin antibody to reflect loading variations. These experiments were duplicated with similar results. (B) Bar graph showing the chymotrypsin-like proteasome activities of lysates prepared from untreated cells or those treated with various proteasome inhibitors, as indicated (*P < 0.05, **P < 0.001 vs. column 1, Student’s t-test). Control refers to lysates added with MG132 in vitro (C) Left, A portion of Triton-X-soluble lysates prepared from untreated or MG132-treated HEK293 cells expressing FLAG tagged parkin alone or with myc-tagged Ubc13 were subjected to anti-myc immunoprecipitation followed by anti-FLAG and anti-myc immunoblotting (IPmyc). The remainder lysates prepared from these variously transfected cells (INPUT) were subjected to anti-FLAG and anti-myc immunoblotting to show the expression levels of FLAG-parkin and myc-Ubc13 respectively. These experiments were replicated at least three times. (D) Anti-K63 immunoblot of lysates prepared from FLAG-parkin transfected MG132-treated cells in the absence or presence of 2 shRNA species to Ubc13 or control shRNA. The blots above were stripped and reprobed with anti-actin antibody to reflect loading variations. The efficiency of the Ubc13 knockdown is shown in the anti-Ubc13 blot. Notice that the level of parkin as revealed by anti-parkin blot is reduced in the presence of Ubc13 silencing. (E) Anti-parkin immunoblot of lysates prepared from FLAG-parkin transduced WT or Ubc13-/- MEFs in the absence or presence of MG132 treatment, as indicated. The blot [file pone.0073235.s002.pdf]

Figure S3

A

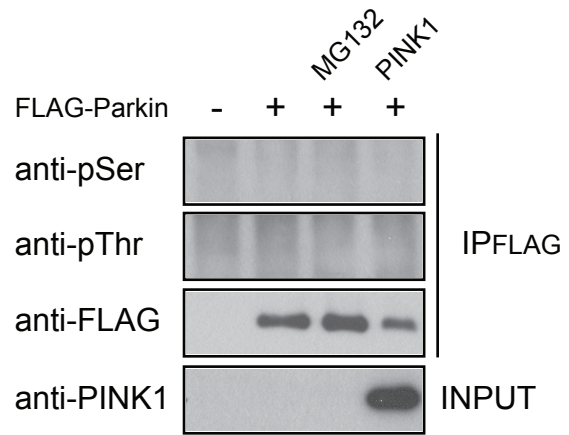

B

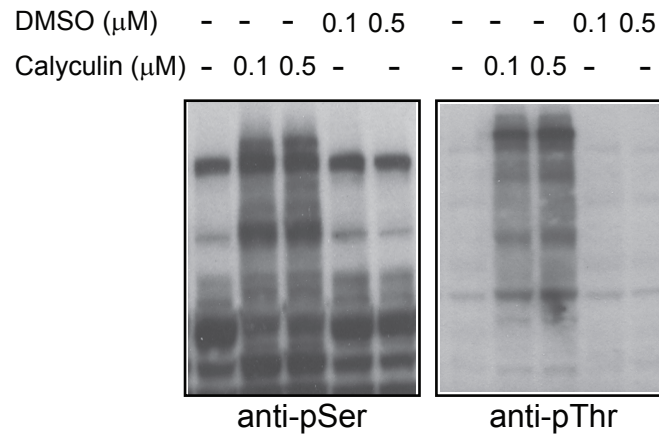

C

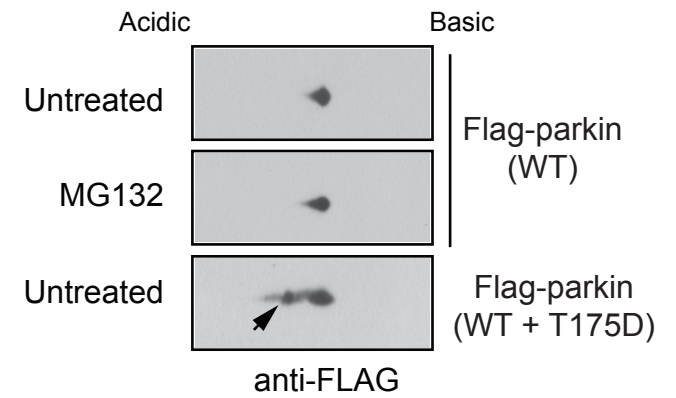

Supplement: Figure S3 — Parkin does not appear to be phosphorylated in the presence of MG132. (A) Anti-phosphoserine and anti-phosphothreonine immunoblots showing the absence of serine/threonine phosphorylation of immunoprecipitated FLAG-tagged parkin in the absence or presence of MG132 treatment or PINK1 co-expression. (B) Anti-phosphoserine and anti-phosphothreonine immunoblots of lysates prepared from cells treated with DMSO or Calyculin a, a potent protein phosphatase inhibitor, shows that the antibodies work fine. (C) Anti-FLAG immunoblotting of 2D gel fractionated cell lysate prepared from FLAG-tagged parkin transfected cells in the absence or presence of MG132 treatment. Note that the parkin-positive spot remains unmodified in both cases (top and middle panels). As a control, when a phospho-mimetic parkin T175D mutant is co-transfected with wild type (WT) parkin, a more acidic parkin-positive spot (arrowhead) can be observed alongside the unmodified one (bottom panel). (PDF) [file pone.0073235.s003.pdf]

Figure S4

A

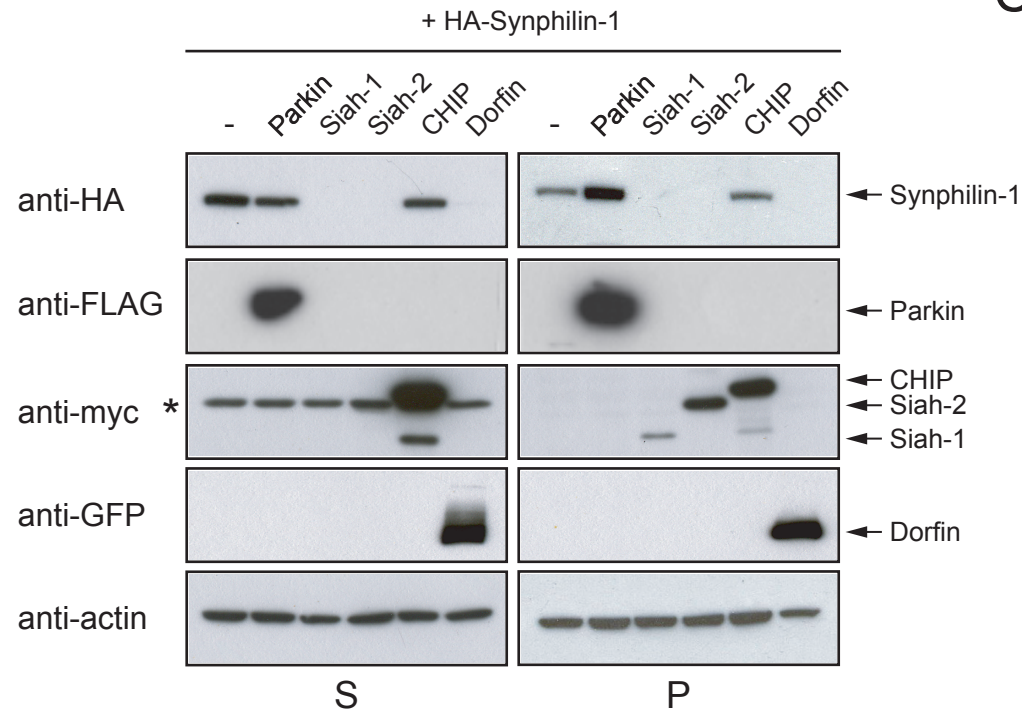

B

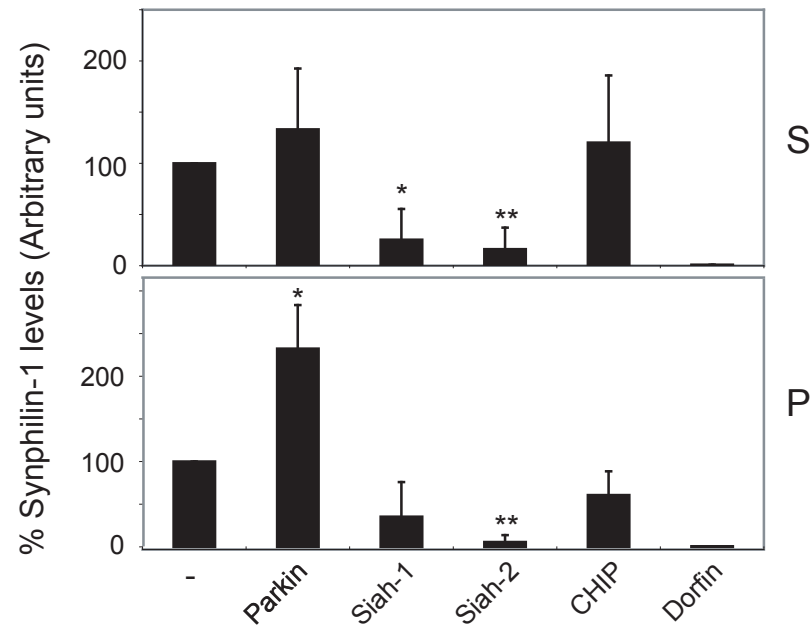

C

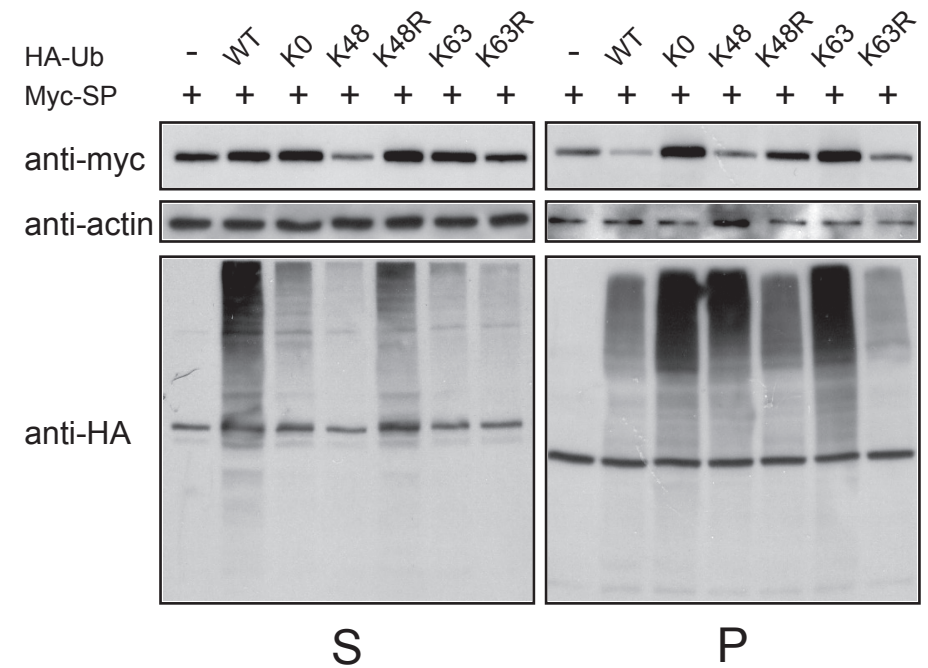

D

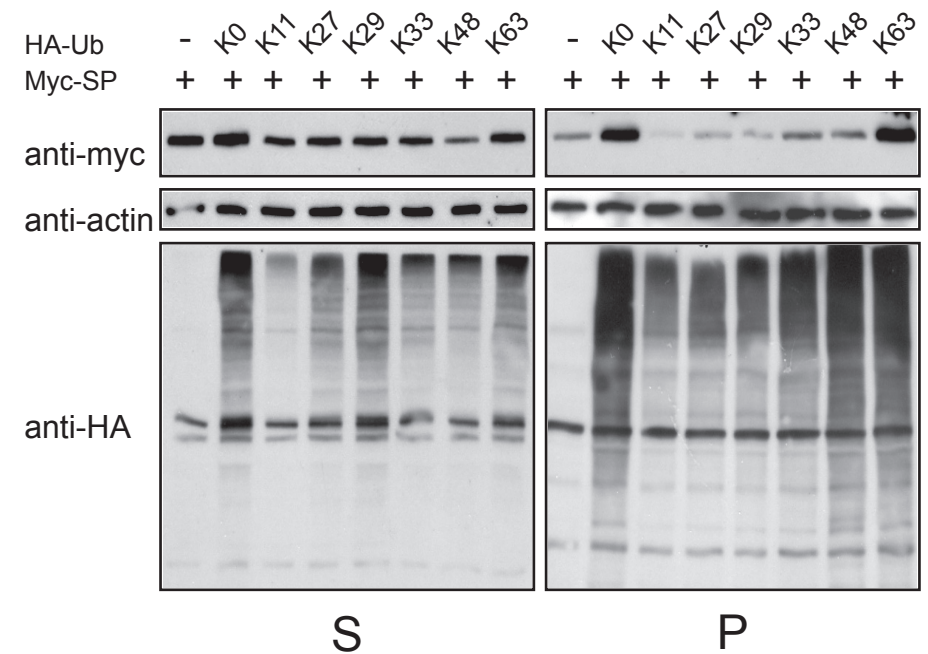

Supplement: Figure S4 — Accumulation of synphilin-1 in cells expressing K63 mutant ubiquitin. (A) Cell extracts sequentially prepared with Triton-X 100 (S) and SDS (P)-containing buffer from HEK cells transfected with HA-synphilin alone or with FLAG-parkin, myc-Siah-1 or -2, myc-CHIP or GFP dorfin were subjected to immunoblotting with various antibodies, as indicated. Asterisk denotes non-specific bands. Equal loading of the different cell lysates was verified by anti-actin immunoblotting. (B) Bar graphs showing the steady state levels of HA-synphilin in S and P fractions of cell lysate after normalization to their respective loading controls (*P < 0.05, **P < 0.001, Student’s t-test). (C–D) Representative anti-myc and anti-HA immunoblots of cell extracts sequentially prepared with Triton-X 100 (S) and SDS (P)-containing buffer from HEK cells transfected with myc-tagged synphilin-1 (Myc-SP) and various ubiquitin species, as indicated. Equal loading of the different cell lysates was verified by anti-actin immunoblotting. (PDF) [file pone.0073235.s004.pdf]

Figure S5

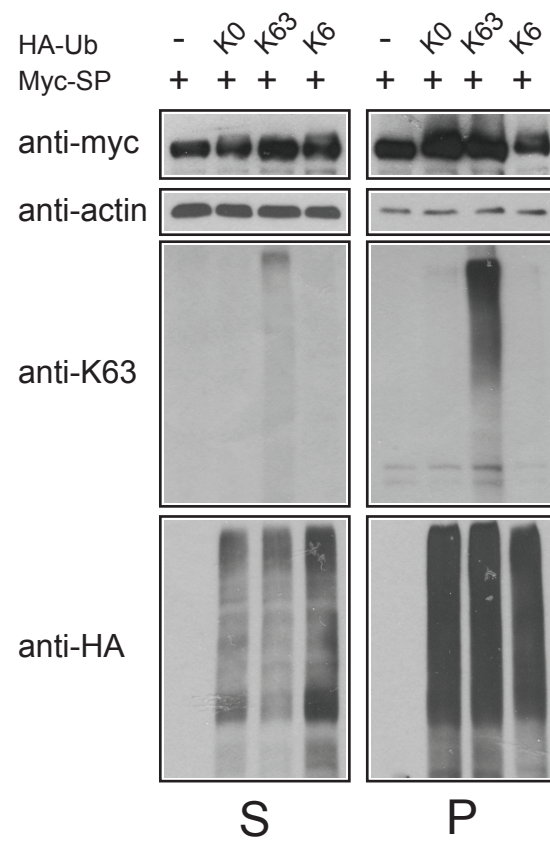

Supplement: Figure S5 — Anti-K63 antibody does not cross react with K0 ubiquitinated proteins. Representative anti-myc, anti-K63 and anti-HA immunoblots of cell extracts sequentially prepared with Triton-X 100 (S) and SDS (P)-containing buffer from HEK cells transfected with myc-tagged synphilin-1 (Myc-SP) and K0, K63 or K6 ubiquitin mutant, as indicated. Equal loading of the different cell lysates was verified by anti-actin immunoblotting. (PDF) [file pone.0073235.s005.pdf]
